# μBialSim: Constraint-Based Dynamic Simulation of Complex Microbiomes

**DOI:** 10.3389/fbioe.2020.00574

**Published:** 2020-06-10

**Authors:** Denny Popp, Florian Centler

**Affiliations:** UFZ – Helmholtz Centre for Environmental Research, Department of Environmental Microbiology, Leipzig, Germany

**Keywords:** microbial communities, metabolic modeling, constraint-based modeling, cross-feeding, microbiome dynamics

## Abstract

Microbial communities are pervasive in the natural environment, associated with many hosts, and of increasing importance in biotechnological applications. The complexity of these microbial systems makes the underlying mechanisms driving their dynamics difficult to identify. While experimental meta-OMICS techniques are routinely applied to record the inventory and activity of microbiomes over time, it remains difficult to obtain quantitative predictions based on such data. Mechanistic, quantitative mathematical modeling approaches hold the promise to both provide predictive power and shed light on cause-effect relationships driving these dynamic systems. We introduce μbialSim (pronounced “microbial sim”), a dynamic Flux-Balance-Analysis-based (dFBA) numerical simulator which is able to predict the time course in terms of composition and activity of microbiomes containing 100s of species in batch or chemostat mode. Activity of individual species is simulated by using separate FBA models which have access to a common pool of compounds, allowing for metabolite exchange. A novel augmented forward Euler method ensures numerical accuracy by temporarily reducing the time step size when compound concentrations decrease rapidly due to high compound affinities and/or the presence of many consuming species. We present three exemplary applications of μbialSim: a batch culture of a hydrogenotrophic archaeon, a syntrophic methanogenic biculture, and a 773-species human gut microbiome which exhibits a complex and dynamic pattern of metabolite exchange. Focusing on metabolite exchange as the main interaction type, μbialSim allows for the mechanistic simulation of microbiomes at their natural complexity. Simulated trajectories can be used to contextualize experimental meta-OMICS data and to derive hypotheses on cause-effect relationships driving community dynamics based on scenario simulations. μbialSim is implemented in Matlab and relies on the COBRA Toolbox or CellNetAnalyzer for FBA calculations. The source code is available under the GNU General Public License v3.0 at https://git.ufz.de/UMBSysBio/microbialsim.

## Introduction

Microbial communities are ubiquitous in nature, thriving in diverse habitats ranging from the deep subsurface (Dutta et al., 2018) over digestive tracts of higher animals (Gould et al., 2018) to the upper troposphere (Deleon-Rodriguez et al., 2013). They are self-organizing entities which both modulate the environment they are embedded in, as well as their own constituents in terms of abundance of individual member populations. Typical natural and engineered microbiomes engage in numerous metabolic and non-metabolic interactions and contain a large fraction of not-yet cultured species. The resulting complexity makes microbiomes notoriously difficult to study. Meta-OMICS techniques help to uncover the metabolic potential and current activity of microbiomes. However, most analyses based on such data remain observational in nature and cannot be used to derive quantitative predictions. The mathematical modeling of microbiomes holds the promise to move from observation to a more quantitative understanding of microbiome dynamics and underlying mechanisms (Song et al., 2014; Widder et al., 2016; Bosi et al., 2017; Succurro and Ebenhöh, 2018).

Focusing on metabolic interaction, a number of dynamic community modeling approaches have been proposed in which activity of individual species is modeled using constraint-based techniques based on genome-scale metabolic network reconstructions (Biggs et al., 2015). Some of these approaches require the definition of a secondary community objective in addition to the standard growth maximization objective for individual species (e.g., d-OptCom; Zomorrodi et al., 2014), a priority list of objectives (DFBAlab; Gomez et al., 2014), or a pre-allocation of compounds to competing species (Chiu et al., 2014). Other models additionally allow for parameter calibration (MCM; Louca and Doebeli, 2015), or for the inclusion of space either simulating populations (COMETS; Harcombe et al., 2014, MetaFlux; Karp et al., 2016) or individual microbial cells following a rule-based approach (BacArena; Bauer et al., 2017). With the exception of the last approach, typically only microbiomes of a few species have been considered in simulations yet. In order to be able to mirror the diversity of natural microbiomes, we developed μbialSim. Our simulator is based on the dynamic Flux-Balance-Analysis approach and does not require the definition of any additional objectives or the pre-allocation of compounds. It allows for the simulation of well-mixed microbiomes of high diversity under batch and chemostat conditions with high numerical accuracy due to a novel numerical integration scheme.

We present three exemplary simulation scenarios covering the complexity range from a mono-culture up to a microbial community containing 773 species. These simulations are intended to demonstrate the capabilities and limits of the code and serve as a starting point for constructing own community models. Using generic and identical parameter values for compound uptake across all compounds and microbial species in the high diversity scenario, the presented simulation results are to be interpreted as generic and not intended for detailed biological interpretation.

## Method

### Overview

In order to simulate the fate and metabolic activity of a microbial community we follow the compartmentalized approach in which activity and growth of individual species is modeled by separate genome-scale metabolic network models following the Flux-Balance-Analysis approach (FBA; Varma and Palsson, 1994). All species have access to a common set of pool compounds. This allows for competition between species as they try to consume the same pool compound and cross-feeding if one species produces a pool compound another is able to use for growth. Instead of restricting analysis to steady state dynamics for which the community composition must be defined as a model input (e.g., Hamilton et al., 2015; Koch et al., 2016), we follow the dynamic FBA approach (Mahadevan et al., 2002) in order to be able to simulate dynamic shifts in microbiomes as a consequence of the system's dynamics. In this approach, the steady-state assumption underlying FBA is assumed to hold true for the duration of the numerical integration step. FBA-computed growth and compound exchange rates are then used to update the state variables of the model which encompass microbial biomass and pool compound concentrations. μbialSim is implemented as Matlab code and relies on either the COBRA Toolbox (Heirendt et al., 2019) or CellNetAnalyzer (von Kamp et al., 2017) for performing FBA computations. This allows for the easy incorporation of FBA models prepared with either software in a community model. Space is neglected in the model, hence assuming a well-mixed environment similar to a well-stirred bioreactor. Both batch and chemostat operation can be simulated. Both compounds and microbial populations can be defined to be part of the bioreactor inflow.

### Mathematical Description

The system state is given by (*C, X*), with *C* = (*C*_1_, …, *C*_*m*_) referring to the concentrations (in mM) of *m* pool compounds present in the bioreactor and *X* = (*X*_1_,…, *X*_*n*_) referring to the abundance (in gDW/L) of *n* microbial populations. For each of these populations, the exchange reactions in their metabolic network model which describe the transport of a metabolite across the cell membrane need to be identified. The selection of the metabolites which are actually coupled to corresponding pool compounds is application specific. For example, on the one hand metabolites assumed never to be growth-limiting can be ignored, while on the other hand, compounds for which experimental data is available should be considered. With *k* the number of coupled exchange reactions for species *j, coupReac*^*j*^ = (*r*_1_, …, *r*_*k*_) records the reaction IDs of the respective exchange reactions, *coupComp*^*j*^=(*idx*_*i*_, …, *idx*_*k*_) the indices of the corresponding compounds in *C, coupSense*^*j*^=(*s*_*i*_, …, *s*_*k*_) the directionality of the exchange reaction with the reaction proceeding in the forward direction indicating metabolite excretion for *s* = 1 and metabolite uptake for *s* = −1, *coupVmax*^*j*^ the maximal uptake fluxes, and *coupKs*^*j*^ the corresponding Monod constants (see below).

The dynamics of the system is then given by two sets of ordinary differential equations. Microbial dynamics for species *j* is given by

(1) $$\begin{array}{l} \frac{dX_{j}}{dt}=\left(X_{j}^{inflow}-X_{j}\right)\frac{q}{V}+\mu_{j}X_{j} \end{array}$$

with microbial concentration in the inflow $X_{i}^{inflow}$ (gDW/L), flow rate q (L/h), bioreactor volume V (L), and specific growth rate μ_*j*_ (1/h). The dynamics of pool compound *i* in the bioreactor is given by

(2) $$\begin{array}{l} \frac{dC_{i}}{dt}=\left(C_{i}^{inflow}-C_{i}\right)\frac{q}{V}+\\ \overset{n}{\underset{\begin{array}{l} j=1,i\in coupComp^{j}\\ with\;i\;the\;k-th\;element \end{array}}{\sum }}coupSense_{k}^{j}\times v_{coupReac_{k}^{j}}^{j}\times X_{j} \end{array}$$

with inflow concentration $C_{i}^{inflow}$ (mM) and flux of the exchange reaction $v_{i}^{j}$ (mmol/gDW/h) which is the *i*-th reaction of the *j*-th species.

The specific growth rates μ and exchange fluxes *v* are derived by solving the FBA problem for each species individually. For this purpose, current compound concentrations in the bioreactor need to be translated to maximal allowable uptake rates. This is commonly done by assuming Monod-type kinetics. For the *i*-th exchange reaction of species *j* which is coupled to pool compound ${coupComp}_{i}^{j}$, the current maximal uptake rate is given by:

(3) $$\begin{array}{l} v_{maxUptake,\;i}^{j}=\;coupVmax_{i}^{j}\frac{C_{coupComp_{i}^{j}}}{coupKs_{i}^{j}+C_{coupComp_{i}^{j}}}. \end{array}$$

### Numerical Integration Scheme

While μbialSim can make use of Matlab solvers for numerically integrating Equations 1 and 2 (options solverPars.solverType and solverPars.solver), the computational cost quickly becomes prohibitive for more complex microbial communities. Instead, we have implemented a novel augmented forward Euler method in μbialSim. The forward Euler method uses the system state at time *t*, evaluates Equations 1 and 2 and uses computed rates to derive the system state at time *t* + Δ*t*, with Δ*t* being the integration step size:

(4) $$\begin{array}{l} X\left(t+\Delta t\right)=X\left(t\right)+\Delta t\times \frac{dX\left(t\right)}{dt},\\ C\left(t+\Delta t\right)=C\left(t\right)+\Delta t\times \frac{dC\left(t\right)}{dt}. \end{array}$$

For syntrophic interactions such as in syntrophic propionate degradation (see Example 2), a compound produced by one species (here: hydrogen), needs to be quickly consumed by the syntrophic partner (here: a methanogenic archaeon) as propionate degradation is thermodynamically only feasible for low hydrogen concentrations. This means that typically, the partner features an effective uptake of the compound with a small *K*_*s*_ value in Equation 3. As consumption can become much faster than production, a very negative rate for hydrogen may result in Equation 2. This can lead to the computation of negative concentrations during an integration step (Equation 4). Similarly, this can also be caused by many species competing for a highly attractive compound. Simply setting negative values to zero in each integration step induces a numerical error. Instead, choosing a smaller integration step size can solve this problem, but might significantly prolong simulation time. Hence, in μbialSim the integration step size is reduced only temporarily whenever this situation occurs in order to avoid numerical error at an affordable increase in computational cost. The time step size is reduced in such a way that the concentration of compound *o* at the next time step is close to its steady-state concentration under the assumption that the production process remains constant. We first identify all species which are either producing or consuming compound *o*. We then compute the current total production rate *p* and the current total uptake rate *u* for the compound by summing across the identified species. Additionally, let *f* describe the current rate of concentration change for compound *o* due to a prescribed flow if a chemostat is simulated. The steady-state condition is then given by *p* = *u* - *f*. Treating *p* as fixed, we find that the right-hand side of this equation depends on the compound concentration *C*_*o*_ when combining Equations 2 and 3:

(5) $$\begin{array}{l} u-f=\\ \underset{j\;is\;a\;consuming\;species}{\sum }|Vmax^{j}|\frac{C_{o}}{Ks^{j}+C_{o}}\times X_{j}-\left(C_{o}^{inflow}-C_{o}\right)\frac{q}{V}. \end{array}$$

Under the assumption that compound *o* is the growth-limiting factor for the second species (i.e., the maximal uptake rate is indeed realized) and that growth remains viable for smaller concentrations, the steady-state concentration $C_{o}^{*}$ for compound *o* can be found by reducing concentration *C*_*o*_ in Equation 5 until *p* = *u*($C_{o}^{*}$) - *f*($C_{o}^{*}$). The time step size Δ*t* which leads *C*_*o*_(*t* + Δ*t*) to be evaluated to $C_{o}^{*}$ can then be computed with the help of Equation (4) to:

(6) $$\begin{array}{l} \Delta t=\frac{\left(C_{o}^{*}-C_{o}\left(t\right)\right)}{\frac{dC_{o}\left(t\right)}{dt}.} \end{array}$$

**Figure 1:**
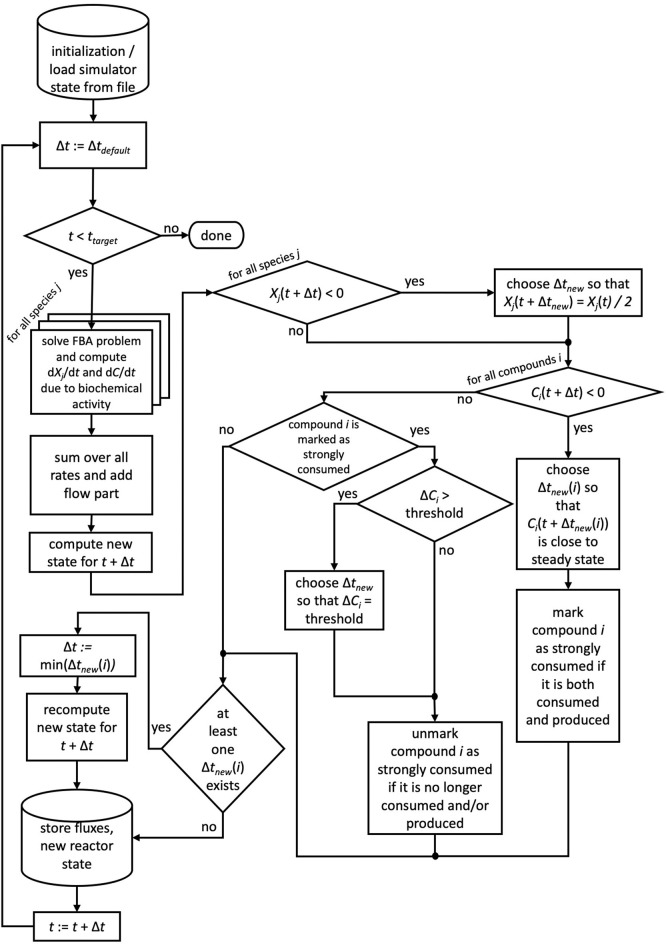
The augmented forward Euler scheme implemented in μbialSim. In each numerical integration step, first the FBA solutions are computed for all member species of the simulated microbiome. The new system state is then computed using obtained rates and the default time step size. If negative concentration values for biomasses or compounds occur, the time step size is reduced as required.

If for more than one chemical compound negative concentrations were calculated using the default time step size, for each of these compounds the described scheme is applied and ultimately the smallest time step size used. We note that reducing the time step size does not require the recomputation of FBA problems, of only Δ*t* changes in Equation 4. For the next time step, the default time step size is restored. Compounds which required the reduction of the time step size are flagged as strongly consumed compounds, as their consumption rate surpassed their production rate. In order to avoid oscillatory behavior for these compounds, μbialSim allows to additionally restrict the time step size in subsequent iteration steps such that the concentration change of these compounds does not surpass a given threshold (parameter solverPars.maxDeviation). If negative biomass concentrations occur, the time step size is reduced such that the biomass concentration is at most reduced by a selectable factor, set to 2 as a default (parameter solverPars.biomassReductionFactor). The flowchart in Figure 1 depicts the complete algorithmic logic of the augmented forward Euler method implemented in μbialSim.

### Features

FBA computations can have non-unique solutions such that different flux distributions lead to the same maximal growth rate. In dFBA simulations, this can cause discontinuities in metabolic fluxes over time. To avoid this, μbialSim implements two features which can individually or in tandem be activated. The first feature is a secondary optimization step which seeks to realize the optimal growth rate as determined by the initial FBA computation, but with minimal fluxes, known as parsimonious FBA (Lewis et al., 2010). This takes into account that high fluxes come with a cost in terms of larger enzyme abundancies and hence should be avoided. The second feature takes a flux distribution and tries to modify it, maintaining the optimal growth rate, to best resemble the flux distribution which was active in the last integration step, a methodology similar to the minimization of metabolic adjustment approach (MOMA; Segrè et al., 2002), which has been applied in the context of dFBA before (Succurro et al., 2019). In contrast to another previous dFBA implementation (Wilken et al., 2018), we do not limit this step to metabolic fluxes crossing the cell membrane, but consider all fluxes to avoid the random flipping of activity on parallel internal pathways between integration steps. In the default settings, both features are activated, leading to three constraint-based computation per model and integration step. First, a regular FBA is performed to identify the optimal growth rate. Second, the absolute sum over all fluxes is minimized while maintaining the determined growth rate. And third, the computed flux distribution is compared to the last integration step and modified to minimize the deviation, maintaining the optimal growth rate, ensuring smooth changes of individual fluxes over time. Simulation results can be stored at each integration step in individual files or in a single result file at the end of the simulation. The former feature (parameter solverPars.recording) is helpful for complex simulations as simulated data are not lost in case of unforeseen server downtimes or other computational calamities. A subsequent simulator run can use the saved data to initialize the simulator and continue the interrupted simulation run (parameter solverPars.readInitialStateFrom).

As loading SBML files and preparing the corresponding data structures can take a while for complex microbiomes, the data structures of the loaded models can be saved as a single file and be used in subsequent simulation runs to speed up initialization (parameter solverPars.saveLoadedModelToFile).

Once the simulation is done, μbialSim computes the overall activity during the simulation for all exchange fluxes of all species (including both exchange reactions which were coupled to pool compounds and those which were not) if desired (parameter solverPars.doMassBalance). This indicates the total compound turnover per species in terms of compound production minus consumption (in mM), and the resulting increase in biomass concentration (in gDW/L). Additionally, three figures to visualize the simulation result are automatically generated. The first figure gives a quick overview over the temporal evolution of all microbial biomass concentrations and all pool compound concentrations over time. In the second figure, all biomass concentrations are plotted in one panel as an offset to the initial biomass concentration, to make dynamics easy to inspect for species having very different initial biomass concentrations, and individual panels for each pool compound. The third figure contains two panels for each microbial species and shows the evolution of coupled exchange reactions, and exchange reactions which were not coupled. Only non-zero exchange fluxes are shown.

For speeding up simulation time, μbialSim can make use of multi-core CPUs (parameters solverPars.parallel, solverPars.maxWorkers). The specified number of Matlab worker processes will be requested at program start and in each numerical iteration step, the FBA problems to be computed will be distributed over the available worker processes.

Two more auxiliary functions are provided to assist in model parametrization and evaluation of cross-feeding patterns. If for a microbial species only an observed maximal specific growth rate μ_*max*_ is known, the function estimateVmax can be used to derive the maximal uptake rate *coupVmax* which leads to the given specific growth rate. For inspecting compound exchange in detail, the function getCmpndExchangeTable can be used that given a simulated trajectory and a specific time generates a table with all coupled exchange fluxes for all species at the specified time. The function filterCmpndExchangeTable can then be used to remove zero entries in this table, or focus on only consumption or production fluxes, or only retain compounds which are at least produced by one species and consumed by at least some other species.

Finally, the option solverPars.recordLimitingFluxes which is activated by default records over time for each species, which fluxes where at their respective upper or lower limit. This option can be used to identify growth limiting compounds.

### Setting Up and Running a Microbiome Simulation

The bioreactor and its operational parameters are defined in the function reactorDefinition_^*^.m. Here, the reactor volume, flow rate, and the list of pool compounds is defined. Additionally, initial concentrations for compounds and biomasses are specified, as well as their concentration in the inflow in case a chemostat is to be simulated.

Loading a FBA model of an individual species of the microbiome to be simulated is recommended to be done in two steps. First, the model is loaded by using the appropriate commands of either the COBRA Toolbox or CellNetAnalyzer in the Matlab function prepareFBAmodel_^*^.m. After loading, if necessary, general constraints on particular reactions can be set, for example to implement a particular scenario. Next, the reaction IDs of the biomass reaction and the non-growth associated maintenance reaction (NGAM) need to be specified. Reaction IDs refer to their running order in the SBML file (or corresponding CellNetAnalyzer data structure). Furthermore, all exchange reactions need to be identified by their IDs and their directionality, that means whether a positive flux indicates compound secretion (Sense = 1) or compound uptake (Sense= −1). Finally, those exchange reactions are identified in the vector IDs which will be coupled to pool compounds present in the bioreactor. The mapping of coupled reactions to reactor compounds is done in the vector reactorCompoundIDs of length *k*, with *k* indicating the number of coupled reactions. The entry at the *i*-th position specifies for the *i*-th coupled reaction, as defined before in the vector IDs, the index of the reactor compound (referring to vector reactor.compounds) to which the exchange reaction is coupled. After this general setup of the FBA model, model parameters are defined in the second step in the function parametrizeFBAmodel_^*^.m. Here, the values for NGAM, and *v*_*max*_ and *K*_*S*_ to define uptake kinetics for all coupled compounds are set.

Finally, the target simulation time, default time step size and other options (see Features) and numerical accuracy parameters are set in the main simulator file microbialSimMain.m.

## Results

Three exemplary simulation scenarios are presented to demonstrate μbialSim's numerical accuracy and performance when dealing with high diversity microbiomes. In all simulations, a reactor of 1 L volume under batch conditions was used (setting *V* = 1 L, *q* = 0 L/h).

### Mono Culture

**Figure 2:**
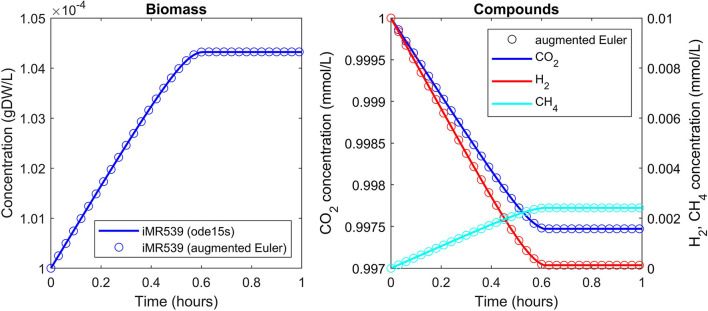
Simulating a hydrogenotrophic batch culture. A *M. maripaludis* population converts H_2_ and CO_2_ to CH_4_ until H_2_ becomes depleted. Both Matlab's ode15s ODE solver (lines) and μbialSim's novel augmented forward Euler method (symbols, every 15th data point is plotted) lead to identical results.

A batch culture of the hydrogenotrophic methanogen *Methanococcus maripaludis* was simulated using the established genome-scale FBA model iMR539 (Richards et al., 2016). The archaeon transforms H_2_ and CO_2_ to CH_4_. Excess CO_2_ was provided such that H_2_ was the growth limiting factor. Model parameters and initial conditions are listed in Table 1. Simulation results show an almost linear growth of *M. maripaludis* until *t* = 0.6 h when H_2_ becomes depleted and growth stops (Figure 2). Simulations using Matlab's ODE solver ode15s and the augmented forward Euler method lead to identical results (Figure 2).

**Table 1:** Model parameters and initial conditions for mono culture simulation.

**Model parameters for** ***M. maripaludis*** **model iMR539**				**Initial conditions**		
**μ (1/d)**	***v_***max***_*^a^ (mmol/gDW/h)**	***K_***s***_* (mM)**	**NGAM (mmol ATP/gDW/h)**	**Biomass (gDW/L)**	**H_**2**_ (mM)**	**CO_**2**_ (mM)**
2.1 (Weinrich and Nelles, 2015)	189.3	4.375 x 10^−4^ (Weinrich and Nelles, 2015)	5.1176 (Richards et al., 2016)	1.0 x 10^−4^	0.01	1.0

a *Was chosen such that the maximal FBA-predicted growth rate matched the specific growth rate μ reported in first table column*.

### Syntrophic Co-culture

**Figure 3:**
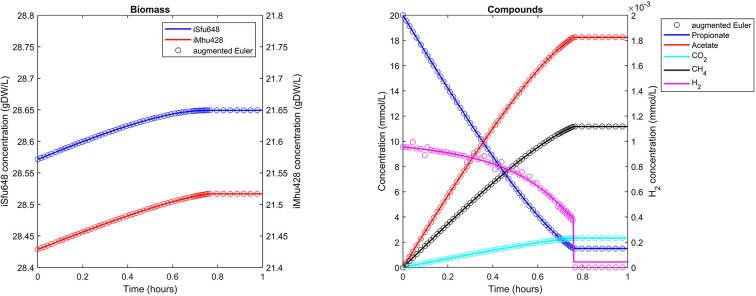
Simulating a syntrophic batch co-culture. Propionate is utilized by *S. fumaroxidans* and converted to acetate, CO_2_, and H_2_. *M. hungatei* then converts CO_2_ and H_2_ to CH_4_. Both Matlab's ode15s ODE solver (lines) and μbialSim's augmented forward Euler method (symbols, every 15th data point is plotted) lead to similar results. As H_2_ is faster consumed than produced, the time step size gets frequently reduced, most notably just prior to the depletion of propionate after which growth of both populations ceases.

The syntrophic conversion of propionate to methane was simulated by using a binary FBA model community of *Syntrophobacter fumaroxidans* (iSfu648) and *Methanospirillum hungatei* (iMhu428) which has previously been simulated at steady state (Hamilton et al., 2015). An initial relative biomass ratio of 3:4 (*M. hungatei*:*S. fumaroxidans*) was chosen as before (Hamilton et al., 2015), and all model parameters are listed in Table 2. Initial compound concentrations were set to 20 mM for propionate, 0.9561 μM for H_2_ and 8.215 μM for CO_2_ which was considered not to be growth limiting for the methanogen. Being produced by *S. fumaroxidans* and quickly consumed by *M. hungatei*, H_2_ was flagged as a strongly consumed compound in the simulation. The time step size became reduced and reached a minimum just prior to the depletion of H_2_ as growth of *S. fumaroxidans* ceased due to low propionate concentrations at *t* = 0.76 h (Figure 3). Except for H_2_, simulation results agreed well if using Matlab's ODE solver or the augmented Euler method. For H_2_, minor fluctuations around the ODE result were apparent at about 0.1 h and between 0.3 and 0.6 h of simulated time when using the augmented Euler method (Figure 3). Most notably, the final H_2_ concentration was 0 instead of the ODE prediction of a final concentration of 43.9 pM. This concentration leads to a growth rate of the methanogen which is just below the threshold below growth is ignored (parameter solverPars.minimalGrowth), and hence no-growth conditions were assumed. A much smaller time step size is required to achieve the same result with the augmented Euler method.

**Table 2:** Model parameters and initial biomass concentrations for syntrophic co-culture simulation.

**Model**	**μ (1/d)**	***v_***max***_*^a^ (mmol/gDW/h)**	***K_***s***_* (mM)**	**NGAM (mmol ATP/gDW/h)**	**Initial biomass (gDW/L)**
*S. fumaroxidans* iSfu648	0.15 (Stams et al., 2005)	1.1738	2.7 (Batstone et al., 2002)	0.14 (Hamilton et al., 2015)	28.57
*M. hungatei* iMhu428	1.2 (Stams et al., 2005)	27.6	0.006 (Stams et al., 2005)	0.025 (Hamilton et al., 2015)	21.43

a *Was chosen such that the maximal FBA-predicted growth rate matched the specific growth rate μ reported in first table column*.

### Human Gut Microbiome

**Figure 4:**
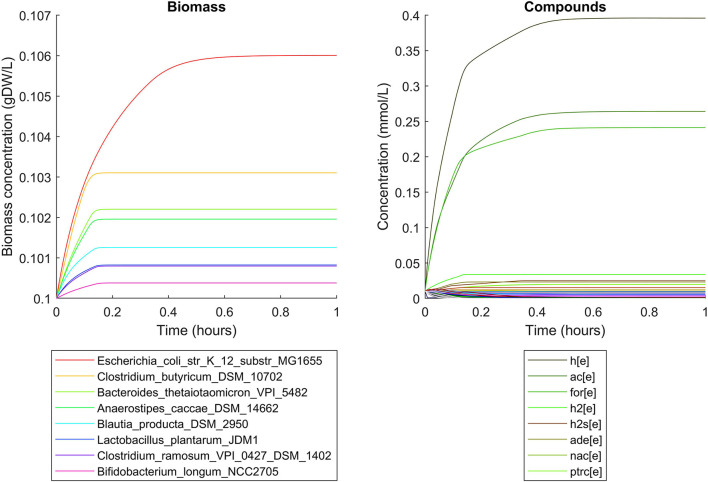
Simulating the SIHUMIx community consisting of 8 microbial species as a batch, using identical initial biomass concentrations for all species and identical compound concentrations for all compounds. The legend for compounds only contains the 8 most abundant compounds (out of 139) at the end of the simulation with abbreviations h[e]: proton, ac[e]: acetate, for[e]: formate, h2[e]: hydrogen, h2s[e]: hydrogen sulfide, ade[e]: adenine, nac[e]: nicotinate, ptrc[e]: putrescine.

**Figure 5:**
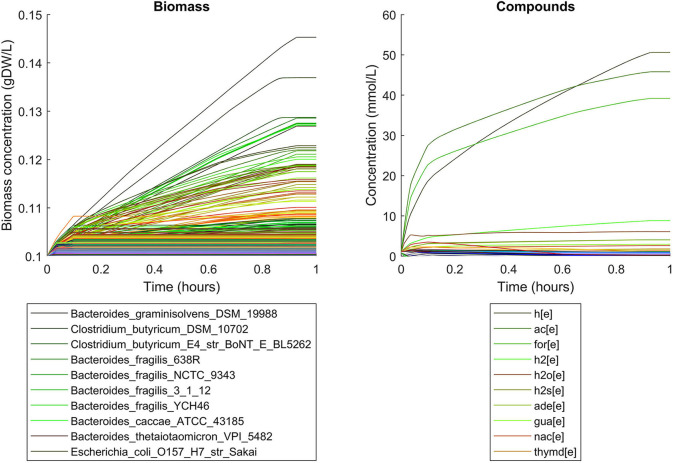
Simulating batch growth of a 773 species gut microbiome with 166 compounds, using identical initial biomass concentrations for all species and identical compound concentrations for all compounds. The legends only account for the 10 most abundant entities at the end of the simulation, with compound abbreviations h[e]: proton, ac[e]: acetate, for[e]: formate, h2[e]: hydrogen, h2o[e]: water, h2s[e]: hydrogen sulfide, ade[e]: adenine, gua[e]: guanine, nac[e]: nicotinate, thymd[e]: thymidine.

**Figure 6:**
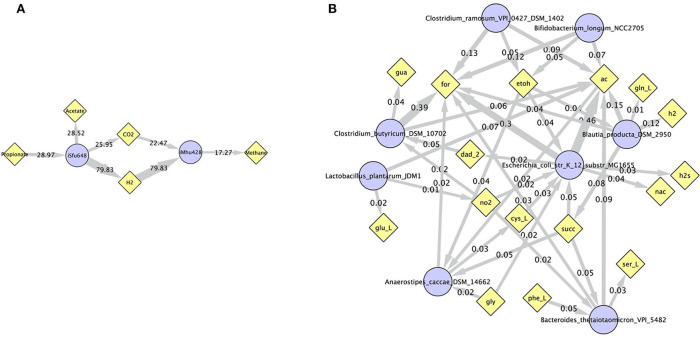
Compound exchange at *t* = 0.1 h for the syntrophic co-culture **(A)** and the simplified human gut microbiome **(B)**. Microbial species (blue circles) produce and consume compounds (yellow diamonds) as indicated by arrows. Numbers indicate production and consumption rates (in mM/h). In **(B)**, only those compounds are shown, which are both produced and consumed by at least one microbial species, and fluxes below 0.01 mM/h as well as compounds H_2_O and proton were omitted for clarity. Abbreviated compound names refer to: ac: acetate, CO2: carbon dioxide, cys_L: L-cysteine, dad_2: 2-deoxyadenosine, etoh: ethanol, for: formate, gln_L: L-glutamine, glu_L: L-glutamate(1-), gly: glycine, gua: guanine, h2/H2: hydrogen, h2s: hydrogen sulfide, nac: nicotinate, no2: nitrite, phe_L: L-phenylalanine, ser_L: L-serine, succ: succinate.

Simulations of the human gut microbiome were based on the AGORA model collection (Version 1.01) comprising 773 microbial human gut species (Magnúsdóttir et al., 2017). Maximal substrate uptake rates (*v*_*max*_) were taken from the individual SBML models, which were configured to mimic a typical western diet (Magnúsdóttir et al., 2017). Exchange reactions in individual models were automatically identified by searching for “EX_” in reaction names. Pool compounds enabling compound exchange were automatically identified by considering those exchange reactions which had at least one flux boundary which was neither zero nor unlimited, hence being provided by the western diet. Monod constants for compound uptake were set to 0.01 mM for all pool compounds. Two different simulations were performed: a simplified human gut microbiome consisting of eight microbial species (see Figure 4 for species list) and 139 pool compounds (SIHUMIx, Becker et al., 2011) and the full collection of 773 microbial species with 166 pool compounds. Batch growth was simulated for 1 h of simulated time. Initial biomass concentrations were set to 0.1 gDW/L for all species, resulting in a total community biomass concentration of 0.8 gDW/L for SIHUMIx and 77.3 gDW/L for the full microbiome. To ensure food consumption within 1 h of simulated time, different initial pool compound concentrations were selected, using 0.01 mM for all compounds in the SIHUMIx simulation and 1.0 mM for the full microbiome, constituting a complex medium which facilitates growth of all species. Indeed, simulations show in both cases, that initially, all species grow. However, as the substrate mix becomes depleted, growth stops for each species at individual times. For the SIHUMIx simulation, *E. coli* has the longest growth phase, stopping at ~0.6 h (Figure 4). In the complex simulation, growth of at least some species is sustained up to almost 1 h (Figure 5). In both simulations, microbial growth is accompanied by the accumulation of acetate and formate (Figures 4, 5). Inspecting compound production and consumption rates for all species at a given time point enables the examination of microbial compound exchange patterns. At 0.1 h into the simulation, compound exchange is straightforward for the syntrophic co-culture (Figure 6A). But already for the simplified human gut microbiome, a complex network emerges (Figure 6B). For 20 compounds, at least one species produces it while another consumes it. The overall largest observed rates are associated with the production of formate and acetate by *E. coli* and *C. butyricum*. Both compounds are produced by all but one microbial species of the community and consumed by the remaining one. Other readily exchanged compounds associated with rates above 0.01 mM/h for four or more species are ethanol, produced by two and consumed by three species and succinate, produced by one and consumed by three species (Figure 6B).

### Performance and Runtime

In order to enable the simulation of microbiomes containing hundreds of species, a novel numerical scheme as an extension to the Euler method was designed and implemented in μbialSim. When comparing simulation runtimes of the presented examples, we find that surprisingly, for the simple examples containing one or two species, Matlab's ODE solver ode15s outperforms the augmented Euler method by up to a factor of three (Table 3). This is likely due to the fact that the augmented Euler method can reduce but not increase the time step size beyond the selected value as the steady state is reached, while Matlab's solver utilizes a fully dynamic time stepping, allowing for large time step sizes as the derivatives become zero. The computational benefit of the augmented Euler method becomes apparent in the eight-species human gut microbiome simulation in which it is more than 50 times faster than the ODE solver. A further speed-up can be achieved by using μbialSims support for parallel computation. In each time step, one FBA computation needs to be performed for each species, which only depends on the current compound concentrations. This allows for an embarrassingly parallel implementation, such that theoretically, at best a linear speed-up in relation to the utilized number of CPU cores can be expected. We find that when using 12 instead of one core in the most complex human microbiome simulation, runtime can be reduced by almost a factor of 8.2 (Table 3). This falls short of the theoretical expectation, but nevertheless brings simulation times into feasible timeframes (from weeks to days), hence enabling for the first time the simulation of microbiomes whose diversity resembles that of their natural counterparts.

**Table 3:** Simulation runtime.

**Example**	**Mono culture**	**Syntrophic co-culture**	**Human gut microbiome**	
**Species**	**1**	**2**	**8**	**773^a^**
ODE solver	1.75 min	7.24 min	31.95 h, parallel (8 cores): 8.69 h	Not tested
Augmented Euler method	3.67 min	22.29 min	0.63 h, parallel (8 cores): 0.22 h	15.40 d, parallel (12 cores): 1.87 d

a *The option to record limiting fluxes was disabled for simulating the 773-species microbiome*.

## Discussion

The simulator code μbialSim significantly expands the microbial diversity which can be addressed in the computational modeling of microbiome dynamics following the constraint-based methodology. However, a number of restrictions apply. For example, a well-mixed system is assumed. Furthermore, the consideration of microbial interactions is restricted to the exchange of growth-related compounds between populations. Even despite these simplifications, the adaptation to experimental data is a challenge, in particular, if microbiomes contain species for which no monoculture data is available. Besides typical constraint-based model parameters to adjust, such as biomass composition and requirements for cellular maintenance, the dynamic simulation calls additionally for the parametrization of the uptake process for all growth-limiting compounds. Two parameters, the maximal uptake rate and the Monod constant, need to be identified for each relevant compound and species. New parameter estimation methods are required to do this efficiently as the complexity in terms of number of parameters and computational demand for forward simulations preclude the application of standard techniques. Our simulator can serve as an invaluable tool during method development. Simulations of arbitrary complexity with known parameter values can provide both the ground truth to compare inference results against, and data of varying density and fidelity as input, enabling a thorough method evaluation. Another challenge is the comparability of simulation output with measured data. While compound concentrations can be readily compared, the model records individual biomass abundancies in gDW/L, which is usually not directly measurable in experiments. PCR-based quantification methods such as amplicon sequencing can provide the required data, but care must be taken during the conversion to a mass-based unit (Bonk et al., 2018). The inclusion of meta-OMICS data to constrain intracellular fluxes for individual species over time is another promising strategy for improving model fit to experimental data. This approach also circumvents the transferability issue of growth behavior in mono and mixed cultures, but faces its own challenges. Once these hurdles are overcome, experimental data from microbiome studies can be directly used to parametrize the simulation model. Such a model will not only provide quantitative predictions regarding microbiome dynamics and activity in response to interventions such as changes in the substrate or bioaugmentation, but will also enable to trace observed behaviors back to their mechanistic causes. This knowledge will inspire novel strategies for directed and precise control of microbiomes in environmental, biotechnological, and medical applications.

Besides its application to measured data, μbialSim can also be used to explore general principles in microbial ecology in a quantitative way, such as substrate competition, facilitation, and the diversity—redundancy relationship. While complex *in silico* microbiomes fall short of a true representation of natural microbiomes due to the discussed limitations, they still capture general features which are likely important driving forces in natural microbiomes. Future versions of μbialSim can additionally consider non-metabolic interactions such as predation, effects of antibiotics, phage dynamics, or host interactions. Furthermore, chemical reactivity of pool compounds could be included as well as a reactor headspace and corresponding gas exchange processes to ease the comparison to experimental data from typical experimental reactor setups. Finally, non-constant chemostat operating regimes could be implemented to facilitate, for example, periodic feeding regimes for simulated microbiomes.

## Data Availability Statement

μbialSim is implemented in Matlab and licensed under the GNU General Public License v3.0 and available for download at https://git.ufz.de/UMBSysBio/microbialsim (or git clone https://git.ufz.de/UMBSysBio/microbialSim.git). Instructions for installation and running the presented examples are provided in the README file.

## Author Contributions

FC designed the simulator and performed the simulations, DP designed scenarios and evaluated simulation results. All authors implemented the code, and prepared and approved the final manuscript.

## Conflict of Interest

The authors declare that the research was conducted in the absence of any commercial or financial relationships that could be construed as a potential conflict of interest.
